# Differences in the Contractile Properties of the Biceps Femoris and Semitendinosus Muscles Throughout a Season in Professional Soccer Players

**DOI:** 10.2478/hukin-2022-0088

**Published:** 2022-11-08

**Authors:** Daniel Fernández-Baeza, Germán Diaz-Ureña, Cristina González-Millán

**Affiliations:** 1 Francisco de Vitoria University Carretera Pozuelo a Majadahonda Km 1.800, 28223 Madrid. Spain

**Keywords:** tensiomyography, hamstring, soccer, injury

## Abstract

The aim of this study was to monitor seasonal changes in the mechanical and neuromuscular characteristics of the knee flexor muscles with tensiomyography, the biceps femoris (BF) and semitendinosus (ST) muscles, of 27 soccer players. All male professional soccer players (age 25 ± 4 years) were measured at the beginning of the preseason (second week) and in the competitive season (10 weeks later). The variables contraction time (Tc) and muscle displacement (Dm) showed significant differences in some muscles, and in others they indicated a tendency to change. In general, the BF improved (more explosive and better muscle tone) and the ST worsened (slower and worse muscle tone) its values during the season. The findings of this study suggest that usual daily soccer training and weekly competition might produce antagonistic changes between the knee flexor muscles.

## Introduction

In all sports, certain physical qualities stand out, demonstrating how to develop such activity in the most suitable way possible and making the most of it during competition. The main components of soccer-specific fitness include explosive strength of the lower extremities, where the knee extensors are involved in running, jumping, and kicking, and the knee flexors are involved in running, influencing the stride length and stabilising the knee joint in change of direction, in acceleration and deceleration, and during landing (Loturco et al., 2016). A high number of intense high-speed actions require the hamstrings to perform in a position of an extreme stretch. The predominant hamstring injury mechanism in soccer occurs during high-speed running or acceleration efforts (Chumanov et al., 2011; Schuermans et al., 2014). The myotendinous junction of the long head of the biceps femoris (BF) is most commonly injured (Ekstrand et al., 2016; Koulouris and Connell, 2003). This means that significant muscle strength imbalances may increase muscular discomfort and result in high injury risk (Junge et al., 2020). Therefore, adequate training and prevention methods are essential to reduce injury risk and decrease the probability of an athlete being unable to participate in training or match play (Carvalho et al., 2016).

The neuromuscular system of soccer players is a key factor in their competitive performance (Read et al., 2019), and its characteristics can be assessed by means of tensiomyography (TMG) (Valencic and Djodjevic, 2001), a non-invasive technique that, by means of a portable device, can measure the properties of individual superficial muscles by recording the isometric muscle contraction induced externally by electrostimulation. TMG can provide information about the muscle fibre type composition, muscle tone, muscle fatigue, and muscle imbalance and asymmetries (García-García et al., 2017; Loturco et al., 2016; Macgregor et al., 2018). This is relevant because it is necessary to conduct regular physiological monitoring of players to assess the effectiveness of training. ^1^ – *Faculty of Health*, *Francisco de Vitoria University, Madrid, Spain*.

However, monitoring of this kind requires knowledge of a player’s initial status, since these reference values of physical and physiological condition are known to change as the season progresses (Turner and Stewart, 2014).

There are very few studies with TMG in soccer players at different phases of the season, and none evaluated the semitendinosus (ST) muscle. For this reason, the aim of this study was to determine the neuromuscular profile of the knee flexor muscles in professional soccer players in two different moments: the preseason and the competitive season. We obtained data of the contraction time (Tc) and muscular displacement (Dm) in the BF and the ST.

## Methods

### Participants

The sample comprised 27 participants, all professional male soccer players from a team in the Spanish professional league (Second Division). All these players (age 25.04 ± 4.50 years, body mass 75.5 ± 7.7 kg, body height 179 ± 6 cm) were in good health and injury-free. All players had more than 15 years of soccer training experience. The team trained 15 to 20 h per week during the preseason, where 6 to 8 h were dedicated to strength development. During the competitive season the team trained 8 to 10 h per week and worked on strength 4 to 6 h per week depending on the competition calendar.

### Ethical statement

All participants provided written informed consent for publication of their details after being informed about the research process and the possible risks of TMG assessment. The research protocol followed the principles of the Declaration of Helsinki regarding biomedical research involving human subjects (18th Medical Assembly, 1964; revised 2008 in Seoul). The study was approved by the coaching staff and management board of the professional soccer team in question, and approval was obtained from a local ethics committee before the study was undertaken.

### Design and procedure

A comparative study was conducted to assess in-season changes in the mechanical and neuromuscular characteristics of knee flexor muscles in professional soccer players. The first assessment was conducted in their club’s medical facilities in the second week of the preseason. The results of this first TMG assessment were used to develop personalised training programmes for each player. The second assessment was 10 weeks later under the same conditions. The two assessments were performed before regular training sessions.

### Measures

TMG was used to measure the radial muscle belly displacement of the long head of the BF and the ST, knee flexor muscles. Measurements were taken under static and relaxed conditions. The knee flexor muscles were measured with the participant in the prone position and the knee joint fixed at an angle of 150º by means of a specially designed wedge cushion. All measurements were taken by two researchers with expertise in the use of TMG: one in charge of controlling the intensity and frequency of the electrical stimulus and the other in charge of directing the placement of the sensor and the participant´s status in a static and relaxed position.

TMG assessments were performed once the participant had been in a relaxed supine position for 5–8 min (García-García et al., 2019). Electrical stimulation was applied with pulse duration of 1 ms and an initial current amplitude of 30 mA, which was progressively increased in 5-mA steps until reaching 110 mA (maximal stimulator output) or until the muscular response did not change despite increasing the intensity of the stimulus. Consecutive stimuli were separated by a rest interval of 10 s. Of the 17 curves recorded for each participant, only the curve with the highest maximum radial displacement was included in the analysis for each muscle assessed. Measures of radial muscle belly displacement were acquired by means of a digital displacement transducer (GK 30, Panoptik d.o.o., Ljubljana, Slovenia) set perpendicular to the thickest part of the muscle belly. The thickest part of the muscle belly was determined visually and through palpation during a voluntary contraction. The self-adhesive electrodes (5 x 5 cm, Cefar-Compex Medical AB Co., Ltd, Malmö, Sweden) were placed symmetrically at a distance of 5 cm from the sensor (Perotto et al., 2006). The positive electrode was positioned above the measurement point and proximally, while the negative electrode was placed below this point and distally. For all measurements, the point of maximum radial muscle belly displacement was determined by obtaining the time-displacement curve for each muscle, as well as on the basis of low-intensity measurements (20 mA) obtained previously by placing the sensor at different points 2–3 mm apart within the area defined by the electrodes, until the exact point of maximum radial displacement was identified. The electrical stimulus was produced by a TMG-S2 (EMF-FURLAN& Co. d.o.o., Ljubljana, Slovenia) stimulator. Each measurement involved recording the following variables of involuntary isometric contraction produced by the electrical stimulus: maximum radial muscle belly displacement (Dm) in mm; Tc as the time in ms from 10 to 90% of Dm; and delay time (Td) as the time in ms from the onset to 10% of Dm.

### Statistical analysis

Mean (+ SD) computations for descriptive analysis were obtained for all variables. In order to verify the normal distribution of the data, a Shapiro-Wilk test was conducted. The variables that had a non-normal distribution were recalculated using log transformation. After that, normal distribution analysis was verified again. A two-way repeated-measures MANOVA was performed to compare the variables. Two factors were defined (time factor and muscle factor). When a significant F-value was achieved, a Bonferroni post-hoc procedure was performed. Small (<0.06), medium (0.06–0.13), and large (>0.14) effect sizes were calculated using Eta partial squared $\left(\eta_{p}^{2}\right).$All statistical analyses were performed using SPSS 24.0, and the level of statistical significance was set at *p <* 0.05.

## Results

### Multivariate analysis

In the right leg, there was a significant difference in the muscle factor (F_(4.18)_ = 6.868, *p* = 0.002, $\eta_{p}^{2}$= 0.604, 1-β = 0.989), the time factor (F_(4.18)_ = 5.077, *p* = 0.006, $\eta_{p}^{2}$= 0.53, 1-β = 0.904), and the time–muscle factor (F_(4.18)_ = 3.26, *p* = 0.035, $\eta_{p}^{2}=0.42,$1-β = 0.719). In the left leg, there was a significant difference in the muscle factor (F_(4.17)_ = 4.517, *p* = 0.011, $\eta_{p}^{2}$= 0.515, 1-β = 0.858). There were no significant differences in the time factor (F_(4.17)_ = 0.439, *p* = 0.779, $\eta_{p}^{2}$= 0.094, 1-β = 0.128) or the time– muscle factor (F_(4.17)_ = 1.636, *p* = 0.211, $\eta_{p}^{2}$= 0.278, 1-β = 0.397).

**Figure 1:**
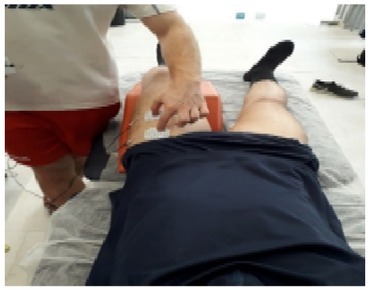
Electrode positioning during measurement by TMG.

**Figure 2:**
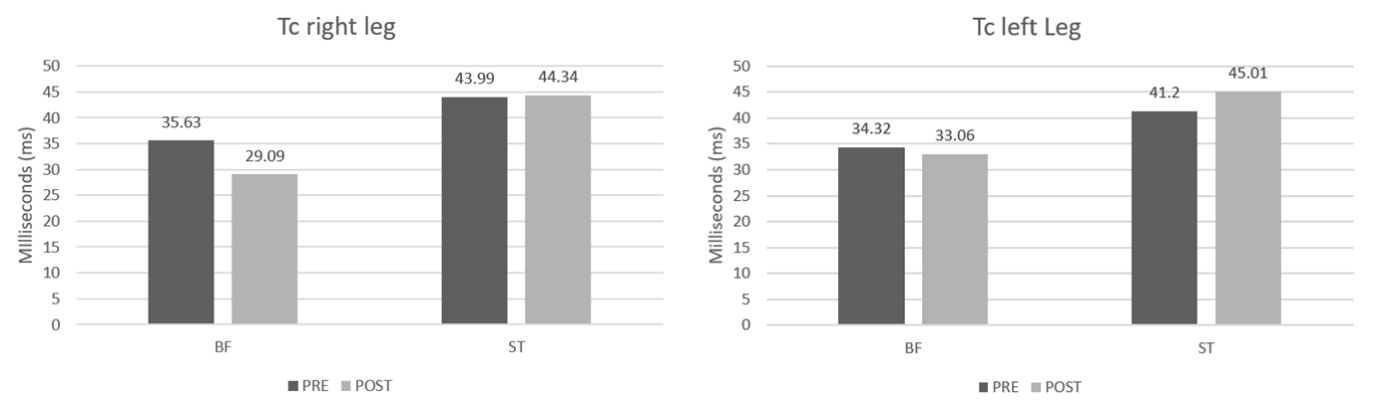
Differences in the contraction time (ms).

**Figure 3:**
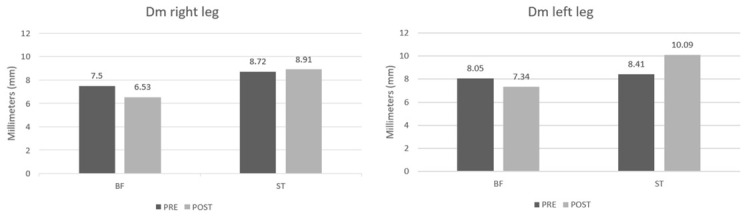
Differences in muscle displacement (mm).

### Univariate analysis

In the muscle factor (M), there were significant differences with a large effect size in the mechanical response in both legs in all variables (Tables 1 and 2). In the time factor (Ti), the right leg showed significant changes in Tc and Td (Table 1), while the left leg did not present significant changes in any of the variables (Table 2). In the time–muscle interaction (TixM) of the right leg, there were significant differences in Tc (*p* = 0.03; $\eta_{p}^{2}$= 0.19). In Dm, although there were no significant differences (*p* = 0.1), there was a trend towards a change with a medium effect size $\left(\eta_{p}^{2}=0.12\right)$In the time–muscle interaction (TixM) of the left leg, there were significant differences in Dm (*p* = 0.02; $\eta_{p}^{2}=$0.23). In Tc, although there were no significant differences (*p* = 0.12), there was a trend towards a change with a medium effect size $\left(\eta_{p}^{2}=0.12\right).$

**Table 1:** Univariate analysis of TMG variables of the right leg.

Variable	Muscle	Pre	Post	*p*			$\eta_{p}^{2}$		
				
				M	Ti	TixM	M	Ti	TixM
TC	BF ST	35_._63 ± 10_._97 43_._99 ± 7_._81	29_._09 ± 12_._95 44_._34 ± 7_._47	>0.01*	0.03*	0.03*	0_._57	0_._21	0_._19

DM	BF ST	7_._50 ± 2_._23 8_._72 ± 2_._21	6_._53 ± 2_._61 8_._91 ± 2_._08	>0.01*	0_._29	0_._10	0_._32	0_._05	0_._12

*Notes: M = muscle; Ti = time; TixM = time–muscle interaction; p = value p;* $\eta_{p}^{2}$*= partial eta square All values in ms, except Dm, which is provided in mm, and VC, which is in mm·s^-1^*.

**Table 2:** Univariate analysis of TMG variables of the left leg.

Variable	Muscle	Pre	Post	*p*			$\eta_{p}^{2}$		
				
				M	Ti	TixM	M	Ti	TixM
TC	BF	34_._32 ± 10_._53	33_._06 ± 12_._45						
	ST	41_._20 ± 8_._87	45_._01 ± 5_._88	>0.01*	0_._54	0_._11	0_._44	0_._02	0_._12

DM	BF ST	8_._05 ± 3_._24 8_._41 ± 2_._21	7_._34 ± 3_._30 10_._09 ± 2_._95	0.049*	0_._43	0.02*	0_._17	0_._03	0_._23

*Notes: M = muscle; Ti = time; TixM = time–muscle interaction; p = value p;* $\eta_{p}^{2}$*= partial eta square All values in ms, except Dm, which is provided in mm, and VC, which is in mm·s^-1^*.

## Discussion

The aim of this study was to determine, by TMG, the variables of Tc and muscle tone in the BF and the ST at two different moments throughout the professional soccer season. The main findings are that the effects of training and competition produced changes in the knee flexor muscles: the BF improved its values, while the ST´s value worsened. In some cases, significant differences were shown; in others, the effect size indicated a tendency to change. The most relevant aspect of this study is that both muscles changed in the opposite way during the season, which seems to show a lack of intramuscular coordination among both hamstring muscles, which worsened through the season.

The time of contraction in ms measured with TMG reflects the explosiveness of the muscle. A muscle with a short time of contraction indicates high explosiveness, and a muscle with a long time of contraction shows a slow muscle (Rodríguez-Matoso et al., 2012; Simunič et al., 2011). The Tc also depends on the percentage of rapid or slow fibres in the evaluated muscle. When the percentage of 11 fibres increases, the Tc decreases (Rodríguez-Matoso et al., 2010, 2012).

Muscular displacement (Dm in mm), measured by TMG, is a variable that reflects muscle tone and stiffness (García-Manso et al., 2011; Rodríguez-Matoso et al., 2015). If the stimulated muscle has great displacement, it indicates a lack of muscular tone; when the muscle has little displacement, it shows stiffness in the muscle. On the contrary, if the muscle`s displacement is adequate, it demonstrates good muscle tone.

In our study, when comparing data from the preseason and the competitive season, values of the Tc (ms) were worse in the ST and higher when the season progressed (right leg 43.99 ± 7.81 ms vs. 44.34 ± 7.47 ms; left leg 41.20 ± 8.87 ms vs. 45.01 ± 5.88 ms). This indicates that the ST muscle turned into a slower muscle when the season progressed. On the contrary, the values of the BF improved throughout the season (right leg 35.63 ± 10.97 ms vs. 29.09 ± 12.95 ms; left leg 34.32 ± 10.53 ms vs. 33.06 ± 12.45 ms), which shows that the BF improved its explosiveness.

The data in our study related to the BF do not concur with the García-García et al.'s (2016) study in which the measurements were conducted at two different moments in the season with TMG. That study indicated that the BF muscle lightly worsened its time of contraction during the season (28.8 ± 5.9 ms vs. 29.8 ± 4.6 ms). The differences between our study and theirs could be related to the time of the first measurement. Our study was performed at the beginning of the season, and the study by García-García et al. (2016) was developed at the end of the preseason, specifically at the beginning of the competitive period, about four weeks later than our evaluation. That is why the data from our study in the first attempt are superior to theirs in Tc. On the other hand, our data coincide with the study by Loturco et al. (2016), where the BF`s Tc also improved (pre: 24.5 ± 10.6 ms, post: 19.6 ± 9.3 ms). That study measured the pre- and post-test values with eight weeks of difference. Regarding the ST`s Tc, our data coincide with the study carried out by Piqueras-Sanchiz et al. (2020) with professional indoor soccer players, in which the ST was shown to lose explosiveness.

Regarding the Dm, which shows the muscle tone, in our data, the BF improved its muscle tone throughout the season (right leg 7.50 ± 2.23 mm vs. 6.53 ± 2.61 mm; left leg 8.05 ± 3.24 mm vs. 7.34 ± 3.30 mm). This coincides with the study by Loturco et al. (2016) with Brazilian soccer players (4.48 ± 1.9 mm vs. 3.04 ± 2.18 mm) in which the BF`s muscle tone also improved. Even though the data of the BF improved in both studies, the study carried out by Loturco et al. (2016) shows that the BF`s muscular displacement in the two measurements was better than ours, probably because the first measurement was made after a brief rest after finishing a national championship and due to the age of soccer players who were two years younger on average. Likewise, our data results do not coincide with the study by García-García et al. (2016), in which the BF`s muscular displacement rose (5.3 ± 1 mm vs. 6.6 ± 1.9 mm), worsening the muscle tone during the season.

Regarding the ST`s Dm, in our study, it worsened during the season, increasing Dm (right leg 8.72 ± 2.21 mm vs. 8.91 ± 2.08 mm; left leg 8.41 ± 2, 21 mm vs. 10.09 ± 2.95 mm), which means that as the season progressed, the ST`s muscle tone worsened. The ST has been measured in few studies (not in practice either), which draws a lot of attention due to its importance. We found only one study in which the measurements were taken at two different moments of the season and that included the ST muscle, it was a study carried out by Piqueras-Sanchiz et al. (2020). Although that study included indoor soccer players, in their measurements the ST also worsened muscle tone during the season (9.7 ± 2.0 mm vs. 10.6 ± 2.0 mm). In the study by Alvarez-Diaz et al. (2016) in soccer players, ST data with little muscle tone were also observed, just like in our study. However, that study did not perform different measurements throughout the season, but rather compared the dominant leg with the non-dominant leg (9.4 ± 2.7 mm and 9.7 ± 2.8 mm, respectively) in a measurement that was made in the competitive period.

What is evident in our study is that both muscles, the BF and the ST, even though they are synergists and share a proximal tendon, present different patterns in muscle Tc and muscle tone. In addition, the most striking thing is that these differences increase as the competitive period progresses: the BF improves its tone and explosiveness, while the ST worsens in both variables. It has been observed that during the sprint there is a greater activation of the BF compared to the ST (Higashihara et al., 2010; Wilmes et al., 2021), specifically greater eccentric activation (Bourne et al., 2014). This seems to be the explanation as to why at the beginning of the competition, when sprints increase in frequency, differences between the muscles also increase: the ST worsens, while the BF improves.

On the other hand, it has been found that, during sprints, the maximum stretch of the muscle–tendon unit (MTU) is higher for the biceps femoris long head (BFLH) compared to the semimembranosus muscle and ST (E. S. Chumanov et al., 2007; Schache et al., 2011). Disparities have also been observed in the magnitude and/or timing of medial versus lateral hamstring activation during running (Higashihara et al., 2010; Silder et al., 2010).

Considering soccer players who have suffered a previous hamstring injury, the lack of muscular coordination between the BF and the ST increases. It has been observed (Schuermans et al., 2014) that the ST loses capacity in its eccentric contraction, and this deficit is compensated by the BF. Furthermore, the shorter length of the bundle of the BF with respect to the ST makes it less tolerant to stretching (Kellis et al., 2012) and makes it difficult to control movement in the final phase of the stride in a sprint at maximum speed. Because of this lack of coordination between both muscles, the risk of injury in both, but more likely in the BF, is high.

Prescribing and monitoring hight-speed running is the most common strategy to reduce the risk of hamstring injuries (Mendiguchia et al., 2020), as sprinting induces greater increases in BF bundle length. However, if there are differences between both muscles, the ST and the BF, in their intramuscular coordination, and these are not corrected before the prescription of repeated sprints, sprinting will increase these differences and, therefore, the risk of injury. On the other hand, before performing sprints, to prevent hamstring injuries, the muscular stiffness of these muscles should be checked, since if their stiffness is very high, the risk of injury with the sprint increases (Alentorn-Geli et al., 2015; Kellis et al., 2012). Therefore, it would be worthwhile to increase the work on the ST to improve intramuscular coordination with the BF and for the load during the sprint to fall equally on both muscles and reduce excessive muscle stiffness, if any (very common in explosive sports), since if the sprint increases the length of the muscle fascicle and it is not prepared for that stretch due to its excessive stiffness, injury is very likely to occur.

A limitation of the study is the difficulty in measuring professional soccer teams, especially at the end of the season. Therefore, more evaluations during the season could give a clearer tendency of contractile properties of these muscles; perhaps at the beginning and the end of the preparation phase and in the middle and the end of the competitive period.

This research demonstrates the importance of evaluating our athletes at different times of the season, to be able to individualise training programmes according to the values obtained in TMG. Likewise, soccer coaches should implement a strength training program with analytical exercises focused on the semitendinosus, to balance the intermuscular coordination elicited by training and competition between the BF and the ST.

## Conclusions

In conclusion, training programmes and competition change the contractile properties of the knee flexor muscles. The BF improves its muscle tone and explosiveness, while the ST worsens both values, which means slower muscle and increased strength deficit during the season measured with TMG. The variables of TMG investigated (Tc, Dm) inform about the reaction and Tc, which is a key factor in soccer, as well as the muscle tone, to determine if a muscle has a deficit in tone or stiffness. These findings are important for coaches who should conduct training programmes with analytical exercises for the ST, monitoring soccer players to avoid injuries and improve performance. Furthermore, with the data obtained from TMG, coaches should individualise training programmes and adjust the adequate training load. The programmes should be re-evaluated throughout the season. Moreover, TMG is a non-invasive technique that does not induce fatigue in the player and does not affect the training programme.

## References

[j_hukin-2022-0088_ref_001] Alentorn-Geli E., Alvarez-Diaz P., Ramon S., Marin M., Steinbacher G., Boffa J. J., Cuscó X., Ballester J., Cugat R. (2015). Assessment of neuromuscular risk factors for anterior cruciate ligament injury through tensiomyography in male soccer players. Knee Surgery, Sports Traumatology, Arthroscopy.

[j_hukin-2022-0088_ref_002] Alvarez-Diaz P., Alentorn-Geli E., Ramon S., Marin M., Steinbacher G., Boffa J. J., Cuscó X., Ares O., Ballester J., Cugat R. (2016). Effects of anterior cruciate ligament injury on neuromuscular tensiomyographic characteristics of the lower extremity in competitive male soccer players. Knee Surgery, Sports Traumatology, Arthroscopy.

[j_hukin-2022-0088_ref_003] Bourne M., Opar D., Shield A. (2014). Hamstring muscle activation during high-speed overground running: impact of previous strain injury. British Journal of Sports Medicine.

[j_hukin-2022-0088_ref_004] Carvalho A., Brown S., Abade E. (2016). Evaluating injury risk in first and second league professional Portuguese soccer: Muscular strength and asymmetry. Journal of Human Kinetics.

[j_hukin-2022-0088_ref_005] Chumanov E., Heiderscheit B., Thelen D. (2011). Hamstring musculotendon dynamics during stance and swing phases of high speed running. Medicine and Science in Sports and Exercise.

[j_hukin-2022-0088_ref_006] Chumanov E. S., Heiderscheit B. C., Thelen D. G. (2007). The effect of speed and influence of individual muscles on hamstring mechanics during the swing phase of sprinting. Journal of Biomechanics.

[j_hukin-2022-0088_ref_007] Ekstrand J., Waldén M., Hägglund M. (2016). Hamstring injuries have increased by 4% annually in men’s professional football, since 2001: A 13-year longitudinal analysis of the UEFA Elite Club injury study. British Journal of Sports Medicine.

[j_hukin-2022-0088_ref_008] García-García O., Cuba-Dorado A., Álvarez-Yates T., Carballo-López J., Iglesias-Caamaño M. (2019). Clinical utility of tensiomyography for muscle function analysis in athletes. Open Access Journal of Sports Medicine.

[j_hukin-2022-0088_ref_009] García-García O., Serrano-Gómez V., Hernández-Mendo A., Morales-Sánchez V. (2017). Baseline Mechanical and Neuromuscular Profile of Knee Extensor and Flexor Muscles in Professional Soccer Players at the Start of the Pre-Season. Journal of Human Kinetics.

[j_hukin-2022-0088_ref_010] García-García O., Serrano-Gómez V., Hernández-Mendo A., Tapia-Flores A. (2016). Assessment of the in-season changes in mechanical and neuromuscular characteristics in professional soccer players. Journal of Sports Medicine and Physical Fitness.

[j_hukin-2022-0088_ref_011] García-Manso J. M., Rodríguez-Matoso D., Rodríguez-Ruiz D., Sarmiento S., De Saa Y., Calderón J. (2011). Effect of cold-water immersion on skeletal muscle contractile properties in soccer players. American Journal of Physical Medicine and Rehabilitation.

[j_hukin-2022-0088_ref_012] Higashihara A., Ono T., Kubota J., Okuwaki T., Fukubayashi T. (2010). Functional differences in the activity of the hamstring muscles with increasing running speed. Journal of Sports Sciences.

[j_hukin-2022-0088_ref_013] Junge N., Morin J. B., Nybo L. (2020). Leg extension force-velocity imbalance has negative impact on sprint performance in ballgame players. Sports Biomechanics.

[j_hukin-2022-0088_ref_014] Kellis E., Galanis N., Kapetanos G., Natsis K. (2012). Architectural differences between the hamstring muscles. Journal of Electromyography and Kinesiology.

[j_hukin-2022-0088_ref_015] Koulouris G., Connell D. (2003). Evaluation of the hamstring muscle complex following acute injury. Skeletal Radiology.

[j_hukin-2022-0088_ref_016] Loturco I., Pereira L. A., Kobal R., Kitamura K., Ramírez-Campillo R., Zanetti V., Cal Abad C. C., Nakamura F. Y. (2016). Muscle contraction velocity: A suitable approach to analyze the functional adaptations in elite soccer players. Journal of Sports Science and Medicine.

[j_hukin-2022-0088_ref_017] Macgregor L. J., Hunter A. M., Orizio C., Fairweather M. M., Ditroilo M. (2018). Assessment of Skeletal Muscle Contractile Properties by Radial Displacement: The Case for Tensiomyography. Sports Medicine.

[j_hukin-2022-0088_ref_018] Mendiguchia J., Conceição F., Edouard P., Fonseca M., Pereira R., Lopes H., Morin J.-B., Jiménez-Reyes P. (2020). Sprint versus isolated eccentric training: Comparative effects on hamstring architecture and performance in soccer players. Plos One.

[j_hukin-2022-0088_ref_019] Perotto A. O., Thomas C. C., Underwood F. B. (2006). Anatomical guide for the electromyographer: The Limbs and Trunk. Physical Therapy.

[j_hukin-2022-0088_ref_020] Piqueras-Sanchiz F., Martínez-Aranda L. M., Pareja-Blanco F., Rodríguez-Ruiz D., García-García Ó. (2020). Evolution of contractile properties of the lower limb muscles throughout a season in elite futsal players. Journal of Sports Medicine and Physical Fitness.

[j_hukin-2022-0088_ref_021] Read P. J., Oliver J. L., Ste Croix M. De, Myer G. D., Lloyd R. S. (2019). A review of field-based assessments of neuromuscular control and their utility in male youth soccer players. Journal of Strength & Conditioning Research.

[j_hukin-2022-0088_ref_022] Rodríguez-Matoso D., García-Manso J. M., Sarmiento S., de Saa Y., Vaamonde D., Rodríguez-Ruiz D., Silva-Grigoletto M. (2012). Assessment of muscle response as a control tool in the area of physical activity, health, and sports. Revista Andaluza de Medicina Del Deporte.

[j_hukin-2022-0088_ref_023] Rodríguez-Matoso D., Rodríguez-Ruiz D., Quiroga M. E., Sarmiento S., De Saa Y., García-Manso J. M. (2010). Tensiomygraphy, utility and methodology in the muscular assessment. Revista Internacional de Medicina y Ciencias de la Actividad Física y El Deporte.

[j_hukin-2022-0088_ref_024] Schache A. G., Blanch P. D., Dorn T. W., Brown N. A. T., Rosemond D., Pandy M. G. (2011). Effect of running speed on lower limb joint kinetics. Medicine and Science in Sports and Exercise.

[j_hukin-2022-0088_ref_025] Schuermans J., Van Tiggelen D., Danneels L., Witvrouw E. (2014). Biceps femoris and semitendinosus - Teammates or competitors? New insights into hamstring injury mechanisms in male football players: A muscle functional MRI study. British Journal of Sports Medicine.

[j_hukin-2022-0088_ref_026] Silder A., Reeder S. B., Thelen D. G. (2010). The influence of prior hamstring injury on lengthening muscle tissue mechanics. Journal of Biomechanics.

[j_hukin-2022-0088_ref_027] Simunič B., Degens H., Rittweger J., Narici M., Mekjavić I. B., Pišot R. (2011). Noninvasive estimation of myosin heavy chain composition in human skeletal muscle. Medicine and Science in Sports and Exercise.

[j_hukin-2022-0088_ref_028] Turner A. N., Stewart P. F. (2014). Strength and conditioning for soccer players. Strength and Conditioning Journal.

[j_hukin-2022-0088_ref_029] Valencic V., Djodjevic S. (2001). Influence of acute physical exercise on twitch response elicited by stimulation of skeletal muscles in man. Biomechanical Engineering.

[j_hukin-2022-0088_ref_030] Wilmes E., de Ruiter C. J., Bastaiaansen B. J. C., Goedhart E. A., Brink M. S., Van Der Helm F. C. T., Savelsbergh G. J. P. (2021). Associations between Hamstring Fatigue and Sprint Kinematics during a Simulated Football (Soccer) Match. Medicine and Science in Sports and Exercise, Ahead of Print (Issue July).

